# Supplementary material for: Are carbon emissions trading and green financial instruments synergistic? -Comprehensive quantitative research based on content analysis
Source: PLoS One. 2024 Mar 7;19(3):e0298601. doi: 10.1371/journal.pone.0298601 (PMC10919601; doi:10.1371/journal.pone.0298601)
Supplement: S1 Data — (ZIP) [file pone.0298601.s001.zip › Data/地级市Z_F工作报告.zip!/╣π╬≈/╙±┴╓╩╨/└┤╫╘╣1⁄2╓┌║┼í╛┬φ┐╦╩2╛▌═°í┐_2012.docx]

一、2011年工作回顾

2011年，是我市经济社会发展经受重大考验、实现重大突破的一年。经济社会发展取得了“八个突破”，实现了“十二五”规划良好开局。

——地区生产总值突破1000亿元，初步核算达1032.47亿元，增长11.1%。

——工业总产值突破1200亿元，达1213.86亿元，增长29.1%。继机械产业、玉柴工业园之后，新增水泥陶瓷、食品健康两个超百亿元产业和北流日用陶瓷工业园、陆川县工业集中区两个超百亿元园区，全市超百亿元产业、超百亿元园区分别达到3个。

——财政收入突破80亿元，达85.86亿元，增长24.5%，增幅排全区第4位；其中一般预算收入增幅排全区第3位。

——农民人均纯收入突破6000元，达6269元，增长18.2%；城镇居民人均可支配收入19590元，增长11.04%；分别比全区平均高1038元和736元，增幅分别排全区第2、第3位。

——招商引资实际到位资金突破400亿元，达431.19亿元，增长29.61%。

——非公经济主体总量突破14万户，达14.02万户。

——本外币各项存款余额突破900亿元，达900.68亿元，增长16.81%。在全国地级市中率先实现县县建立村镇银行。

——森林覆盖率突破60%，达60.08%。

主要做了以下工作：

（一）着力统筹城乡发展，一体化发展更加协调。开展镇村规划集中行动，完成5个县域镇村体系规划、14个乡镇总体规划和940个行政村村庄规划。加快“三个集中”步伐，全市新增入园企业121家，园区工业集中度87.67%；新增农村土地流转面积11.15万亩。农村基础设施不断完善，农村公路建设里程1120公里，新增120个行政村通硬化路，行政村通硬化路达80.6%；水利建设投入12.6亿元；解决45.34万人饮水安全问题。建成64个村级公共服务中心、756个村级卫生室、332个村级计生健康服务室；新农合参合率达97.17%。

（二）着力抓好项目建设，发展基础更加坚实。重点推进831个重大项目建设，完成投资410亿元。列入自治区统筹推进的32个重大项目实现了开工率、竣工率和完成前期工作节点任务“三个全区第一”。完成全社会固定资产投资788.82亿元，增长31.4%。广西银亿公司硫磺和石膏制取硫酸等185个重大项目竣工投产。玉林至铁山港高速公路和铁路等项目加快推进。开工建设岑溪南渡至陆川和贵港木格至博白水鸣公路、大容山风电场等290个重大项目。新华联50万吨精制镍铁及深加工等项目前期工作进展顺利。获得中央和自治区支持项目946个，资金17.99亿元。

（三）着力推动企业升级，工业结构更加优化。深入实施“强柴兴玉”战略，玉柴集团销售收入达428.71亿元，率先研发出我国首台符合欧六排放标准的车用柴油机，代表我国内燃机行业最高水平的高效节能环保内燃机国家工程实验室落户玉柴。完成工业投资448.92亿元，实施自治区千亿元产业科技攻关重大专项10项，启动建设清华科技园玉林分园。新增规模以上工业企业140家。实现规模以上工业增加值296.62亿元。

（四）着力发展现代农业，“三农”基础更加牢固。大力实施农业产业“123工程”和农户“万元增收工程”，全市完成农林牧渔业总产值360.42亿元。狠抓“米袋子”、“菜篮子”工程，粮食稳定增产，总产量达178.4万吨；畜禽、水果、蔬菜产量全线增长。新增国家级农业标准化示范区3个、国家级重点龙头企业1家，建设自治区级农业良种培育中心4家，自治区级农业标准化生产技术示范基地7个。

（五）着力扩大城乡消费，现代服务业更加繁荣。大力推进十大商贸物流项目规划建设，北流国际陶瓷贸易城、玉林机电设备物流一期等项目竣工投产，玉林国际商贸城、宏进农批市场二期、交通物流园等项目开工建设。全年商业设施及市场建设投入21.6亿元。全市社会消费品零售总额362.81亿元。旅游总收入68.08亿元。

（六）着力加快城镇建设，城市功能更加完善。制定出台推进城镇化跨越发展的7个配套文件，完成《玉东新区城乡经济社会一体化发展总体规划》等10项规划。坚持以新区开发带动旧城改造，秀水大道建成通车，狮子山公园、龟山公园三期建成开放，文体路、湿地公园、玉柴广场、文化艺术中心等项目加快推进，玉林城区面积扩展到61.6平方公里，人口增加到60万人，绿化覆盖率达36%；投入资金50亿元加快城镇建设。

（七）着力促进全民创业，民营经济更加活跃。市财政安排500万元创业资金，建立创业指导站151个，在全市掀起全民创业热潮。全市发展微型企业3396户，注册资本金3.28亿元，新增创业就业人数3万多人。

（八）着力深化改革开放，发展动力更加强劲。建成市公共资源交易中心并投入运行。完成乡镇机构改革和市县文化市场综合执法体制改革。国家基本药物制度和医疗卫生机构综合改革实现基层全覆盖。完成集体林权主体改革并通过自治区验收。积极推进行政审批改革和扩权强县，市直部门行政审批比2008年减少110项，下放或委托17项。全面参与广西北部湾经济区和西江经济带开放开发，龙潭产业园建设发展步伐加快。成功举办第八届玉博会和第三届药博会，获评为中国最具影响力节庆城市、中国最具投资价值城市。深化玉台合作，台资企业累计达53家，总投资额43亿元。玉柴集团增资7700万美元境外上市获国家发改委核准，成为广西“走出去”标志性项目。

（九）着力推进绿色发展，生态环境更加优良。坚定不移淘汰落后产能，全市拆除落后水泥、造纸生产线49条，淘汰落后产能220.87万吨。规模以上万元工业增加值能耗下降7.3%，万元GDP能耗下降2.5%。城镇污水集中处理率和垃圾无害化处理率分别达95.73%和97.09%。坚持每月“绿化日”植树制度，积极实施“绿满八桂”造林绿化工程，大力创建国家森林城市，全市植树造林19.06万亩。

（十）着力提升公共服务，民生保障更加有力。全市投入各项社会事业发展和民生领域的资金达118.4亿元。参加广西第十二届运动会获得金牌数创历史新高。全市专利申请量和发明专利授权量分别增长43.37%和160%。顺利完成三年医改工作任务，在全区率先完成村卫生室规范化建设。城镇登记失业率控制在4.3%以内。企业职工养老、城镇基本医疗、失业、工伤、生育保险参保人数达154.59万人次。新建145个五保村。开工建设保障性住房1.12万套，新增发放租赁补贴7013户，完成1万户农村危房改造。发放农机购置补贴资金4526万元。推进水库移民新村建设项目59个，发放扶持资金1.49亿元。建成革命老区、贫困村屯级道路204条。

（十一）着力创新社会管理，社会环境更加和谐。加大治安防控体系建设力度，社会治安持续好转，“两抢一盗”等多发性街面犯罪活动明显下降，玉林市和博白县获全区“清网行动”一等奖。市、县、镇、村四级综治信访维稳工作网络进一步完善，“大排查、大接访、大调解、大防控”活动有效推进，信访总量和群体性事件持续下降。

（十二）着力加强政府建设，政府工作更加高效。人大代表议案、建议和政协委员提案100%办结。圆满完成市、县、镇政府换届工作。重大决策机制不断完善，依法行政水平进一步提高；政府绩效管理有效推进。政务服务网络进一步健全，政务公开取得新进展。

二、2012年工作总体要求和目标任务

2012年政府工作的总体要求：以邓小平理论和“三个代表”重要思想为指导，深入贯彻落实科学发展观，紧紧围绕市第四次党代会提出的“统筹城乡发展、推进富民强市、建设幸福和谐玉林”的战略部署，以科学发展为主题，以加快经济发展方式转变为主线，坚持稳中求进、好中求快，深入推进经济结构战略性调整，加快新型工业化、城镇化、农业现代化互动发展，千方百计稳增长、转方式、控物价、惠民生、抓改革、促和谐，保持和扩大经济社会发展良好势头，全力创建非公经济示范市、现代农业示范市、东部产业转移示范市、岭南特色文化示范市、生态文明示范市和社会管理创新示范市，为实现全面建设小康社会目标打下坚实基础。

今年全市经济社会发展主要预期目标是：地区生产总值增长10%，财政收入增长l5%，全社会固定资产投资增长20%，规模以上工业增加值增长20%，社会消费品零售总额增长17%，外贸进出口总额增长20%。万元地区生产总值能耗下降3.89%，化学需氧量、二氧化硫、氨氮、氮氧化物排放量削减控制在自治区下达指标以内。城镇居民人均可支配收入增长11%，农民人均纯收入增长13%。城镇登记失业率控制在4%以内，居民消费价格指数涨幅控制在4%左右。人口自然增长率控制在自治区下达指标内。

三、2012年工作安排

主要抓好以下重点工作：

（一）全面统筹城乡发展，构建城乡一体化发展新格局。围绕统筹城乡“五年大发展”目标，大力推进城乡一体化发展，争当广西统筹城乡发展排头兵。

推进“三个集中”。抓好园区基础设施建设和招商引资，大力推进工业向园区集中，力争今年新增入园项目190个以上。完善县乡农村土地流转服务体系，引导土地向规模经营集中，力争新增农村土地流转面积15万亩。深化农村土地综合整治，加快撤村建居和社区建设，促进农民向二、三产业转移。

实施“三大工程”。坚持城乡风貌改造、城乡清洁、城乡绿化“三大工程”有机结合。加强城乡道路、排水、污水和垃圾处理等基础设施建设。建立完善城乡环境治理长效机制。大力开展植树造林活动，抓好城镇、村屯、交通干道、河堤等绿化建设，努力实现城乡绿化、道路硬化、环境美化。

完善“三大体系”。扎实推进公共财政体系、公共服务体系、社会管理体系建设。

做强县域经济。深入推进扩权强县工作，推动一批管理权限细化落实到县一级政府，扩大县域发展自主权。加强县域分类指导，大力发展特色产业，打响特色产业品牌，促进县域经济跨越发展，力争更多县（市、区）进入广西科学发展“十佳”县行列，争创“西部百强县”。

（二）加快工业转型升级，力求实体经济发展取得新突破。坚持工业发展主导地位不动摇，大力实施“实体经济年”活动，加快推进工业园区建设和产业转型升级，以新型工业化为先导做大做强做优实体经济。

加快工业产业体系建设。深入实施千百亿元产业工程，加快发展壮大机械产业和水泥陶瓷、食品健康、服装皮革、电子信息、有色金属等产业，大力推进玉柴船用柴油机等一批产业项目建设。把加快发展战略性新兴产业作为产业结构升级的重点，大力培育发展新能源、新材料、先进装备制造等新兴产业，加快推进玉柴厂区连片太阳能并网发电等一批项目建设。

大力推进新型装备制造示范基地建设。把建设新型装备制造示范基地作为做大做强做优工业、推动产业转型升级的重大举措。制订扶持政策体系和发展规划，坚持以先进技术、新型产品、新兴产业、绿色环保为主攻方向，大力推进内燃机等传统装备制造业转型升级，做大做强；积极培育发展新型电工电器、新型电子信息设备等新兴装备制造业。加快玉柴工业园、陆川县工业集中区等园区建设，打造“中国绿色动力发动机基地”和“工程机械制造基地”。

继续实施“强柴兴玉”战略。制定完善打造千亿玉柴的扶持政策，大力支持玉柴发展提升核心技术和强优产品，重点抓好玉柴铸造中心、玉柴集团国家级技术中心创新能力建设等重大项目。

全面实施“抓大、壮小、扶微”工程。实施“亿元工业企业工程”，通过项目建设、技术改造、兼并重组、招商引资等途径，培育壮大一批亿元以上企业；力争今年新增亿元工业企业30家。实施“小型企业上规模工程”，引导推动58家主营业务收入2000万元以下的小型企业发展为规模以上企业。实施“扶持微型企业发展工程”，大力发展微型企业，使全市微型企业总数达到7000户左右。

加大工业投入力度。大力推进产业关联度大、技术水平高、市场前景好的工业项目，加快传统优势产业升级改造，全年完成工业投资600亿元以上。

加强工业园区建设。创新园区建设管理体制，积极组建工业投融资平台，加大园区建设投入。坚持区城同建，完善园区配套服务设施，改善园区面貌，塑造园区形象，提升园区竞争力。加强园区土地储备管理。今年全市园区建设计划投入220亿元以上，园区工业总产值突破1000亿元，园区工业增加值达到280亿元。

加大企业自主创新和技术改造力度。推进千亿元产业重大科技攻关工程。大力支持高效节能环保内燃机国家工程实验室、国家内燃机及零部件产品质量监督检验中心、国家级陶瓷检测重点实验室建设，积极提升玉柴股司技术中心等技术中心的研发能力，全面增强自主创新能力。加快中小企业科技创新孵化服务中心建设，培育高新技术企业，争取年内全市高新技术企业达到10家。引导和扶持企业加快设备更新改造，今年技改投资完成380亿元以上。

（三）积极发展现代农业，推动农业现代化取得新进步。全面落实强农惠农富农政策，加快推进农业科技创新，持续增强农产品供给保障能力，壮大农业实体经济，全力创建现代农业示范市。

毫不放松抓好粮食生产。强化粮食生产责任制，抓好整县整乡推进粮食高产示范片建设，全年粮食播种面积稳定在487万亩，力争粮食稳定增产。严格保护耕地和基本农田。

千方百计增加农民收入。实施农户“万元增收工程”，培育一批万元片区、万元村屯和年纯收入10万元以上的“示范农户”。

发展优势特色产业。实施农业产业“123工程”，加快发展壮大畜牧水产等特色产业，建设广西特色农业基地。大力发展有机农业和品牌农业。加大对农业龙头企业的扶持力度，积极发展农民专业合作社。

积极发展林下经济。因地制宜推广林药、林花、林菌等模式，做大做强林下种植、养殖和林下产品加工、林下旅游等产业，建设一批特色林下经济基地。

大力推进农业科技创新与服务体系建设。以海峡两岸农业合作试验区为平台，完善农业科技创新和应用服务体系，辐射带动全市农业科技创新和良种良法推广。推广农作物“三免”、“三避”等先进技术，实施超级稻推广、秋冬种开发和间套种技术推广“三个百万亩行动计划”。推进村庄综合整治，加快农村污水垃圾处理设施和公共服务设施建设，改善农村生产生活环境。

（四）着力扩大消费需求，带动现代服务业上新水平。把扩大消费需求作为推动经济增长的重大举措，完善城乡流通服务体系，活跃城乡消费，壮大服务业实体经济，加快提升现代服务业。

积极扩大消费需求。加强城乡市场流通体系建设，继续实施“万村千乡市场工程”和“新网工程”。大力开展美食节、旅游节、汽车展、商品展等主题促消费活动。扩大商品销售等消费。加强商业信用建设，强化监管和服务，坚决打击商业欺诈、制假售假行为，让广大群众方便消费、放心消费、安全消费。

大力发展商贸物流业。加强“全国流通领域现代物流示范城市”建设，按照“大建市场、建大市场，大建物流、建大物流”的要求，加大市场培育力度，加快推进玉林国际商贸城、宏进农批市场二期、交通物流园等项目建设，力争开工建设玉林“无水港”、红星美凯龙玉林家居商城等项目。

加快发展生产性服务业。把发展生产性服务业作为扩大消费需求、提升服务业的重点。大力发展会展业，办好第九届玉博会、第四届药博会等展会，提升中国（北流）国际陶瓷博览会等展会水平。大力发展信息服务业，加快“无线城市”、“数字城市”、“智慧城市”建设，提高城乡信息化水平。完善科技服务体系。

积极发展现代金融业。继续推进“引银入玉”，力争年内引进1家以上金融机构。加快农村金融改革，培育发展农商行和市级村镇银行。

加快发展旅游业。大力推进容县绣江两岸文化旅游景观、兴业天外天等旅游项目建设，提升大容山国家森林公园等景区景点，加快建设云天文化旅游、玉东都市生态旅游等六大旅游产业集聚区。

（五）突出抓好项目建设，力争项目投资再上新台阶。优化投资结构，提高投资质量和效益，保持投资较快增长，确保今年全社会固定资产投资增长20%，力争突破1000亿元。

统筹推进重大项目开竣工。突出抓好工业项目建设，统筹推进一批生产性服务业、基础设施、民生和社会事业等重大项目。市层面统筹推进重大项目860个，年度计划投资460亿元；其中新开工重大项目280个，年度计划投资220亿元，开工建设新华联50万吨精制镍铁及深加工、玉林嘉德发动机连杆生产等项目；续建重大项目380个，年度计划投资240亿元，抓好福绵国际服装贸易城等项目建设；推进前期工作重大项目200个，抓好玉林市太阳能光伏电池铸锭切片等项目前期工作。计划竣工投产重大项目150个。

着力推进交通基础设施建设。今年计划完成交通固定资产投资38亿元以上，实现县县通高速公路、85%以上行政村通硬化路、93%以上行政村通客车。

加快推进水利设施建设。抓好防汛防洪、病险水库除险加固、农村居民饮水、中小河流治理、小型农田水利建设等工作，今年完成水利水电固定资产投资10亿元以上。

加强能源建设。加快城市电网建设，投入资金5.86亿元，建设500千伏变电站1座、220千伏变电站5座，110千伏变电站10座，竣工投产变电站6座。

扎实做好项目前期和储备工作。加强重大项目的研究、策划、申报等前期工作，不断充实项目储备库。完善市县领导联系重大项目制度和部门并联审批制度，解决好项目前期工作中遇到的问题。

（六）大力加快城镇建设，迈出城镇化跨越发展新步伐。今年计划完成城镇市政基础设施固定资产投资46亿元，力争城镇化率提高2.3个百分点。

加快推进城市新区建设。推进产业园区规划与新区规划有效衔接，加快玉东新区、玉柴新区、龙潭新区建设发展。玉东新区围绕打造现代化城市新区和统筹城乡发展的样板，加快发展高新技术产业、现代服务业和现代农业，高起点、高标准、大手笔推进一批现代化、标志性设施建设，树立现代化城市新形象。积极筹建玉柴新区，加快新型装备制造示范基地建设，完善新区基础设施，建设工业经济和城市建设同步互动发展的动力新城。

加快县（市）城区和重点镇建设。重点建设“兴业县城－玉林城区－北流城区－容县县城”城镇带、“玉林城区－博白县城－龙潭镇”城镇带、“玉林城区－陆川县城－文地镇”城镇带、“北流城区－隆盛镇－清湾镇”城镇带。

促进房地产平稳健康发展。规范房地产市场秩序。

（七）深入推进全民创业，力促非公经济不断有新发展。把壮大非公经济作为富民强市的重大举措，深入推进“全民创业年”活动，先行先试大力发展微型企业，全力创建非公经济示范市。

完善非公经济发展政策机制。建立完善促进非公经济发展的市场准入、税收减免、财政补贴、金融支持等政策体系。坚持非禁即入，深化国有（集体）企业产权制度改革，让更多的非公资本进入。鼓励民间资本参与交通、水利、政策性住房等基础设施和教育、文化、卫生等社会事业建设发展。

培育壮大创业主体。大力引导和支持在外务工、经商和创办企业的成功人士回乡创业。鼓励和引导大中专毕业生自主创业。支持科技人员利用科技成果创办、领办科技初创型企业。扶持农业种养能手扩大生产经营规模，创办农业企业。引导私营企业家进行“二次创业”，培育一批企业家领军人物。积极发展各种行业协会、合作社及销售公司等中介服务组织。

完善创业平台。大力推进创业孵化基地和创业园建设。加强创业指导服务中心和创业培训基地建设，积极开展创业培训。建立完善创业融资平台，鼓励金融机构推出适合全民创业的金融产品，大力发展中小企业融资性担保服务和小额贷款服务。搭建创业信息平台，研究开发适合全民创业的项目，建立创业项目信息库；在政府网站设立全民创业电子窗口，免费为创业者提供政策、市场、项目和科技信息，开展产品推介等服务。

大力扶持发展微型企业。实施“扶持微型企业发展工程”，市财政安排创业资金和微型企业发展资金1200万元，对初始创业者和新设立的微型企业进行创业培训、资本金补助等扶持。今年计划扶持发展微型企业3600户。

（八）坚持深化改革开放，增添科学发展新动力。坚持把改革开放作为加快发展的根本出路，全面推进各领域改革，积极扩大开放合作，全力创建东部产业转移示范市。

加大改革攻坚力度。鼓励和推进企业重组。加强国有企业和国有资产管理。积极改革创新城乡居民户籍管理制度。稳步推进资源性产品价格和环保收费改革。积极探索矿山探矿权、采矿权交易收费改革。深化农村综合改革，引导土地承包经营权依法有序流转。

加强区域合作。全面融入广西北部湾经济区开放开发，启动铁山港东岸玉林产业园开发建设，加快龙潭产业园建设发展，推进广西银亿等企业扩大产能，加快再生资源园区等重大项目建设，做好广西华电玉林热电联产、锂离子电池材料产业基地等项目前期工作，大力培育发展临海产业。积极参与西江经济带建设，抓好绣江复航、玉林至贵港城际铁路等项目前期工作。加强玉台农业、经贸、旅游等方面的合作，提升玉台合作水平。

大力建设承接产业转移示范带。积极参与桂东承接产业转移示范区建设。加快规划建设承接产业转移走廊，以北流日用陶瓷工业园、容县经济开发区为平台，以玉容一级公路为主线，坚持区城同建，加强产业配套，打造承接产业转移示范带。

全面扩大对外开放。用好用足中国-东盟自由贸易区优惠政策，深化以东盟为重点的多区域合作。充分发挥口岸作用，积极开拓海外市场。加强机械机电、日用陶瓷、编织工艺品、皮革及制品等重点企业和行业的跟踪服务，引导外贸企业转型升级。

加强财税工作改革创新。认真落实积极财政政策，进一步培植财源，做大财政“蛋糕”。强化依法治税，加强税收征管，提高收入质量，确保全年财政收入突破100亿元。完善公共财政体系。深化预算改革，严格预算执行管理，扩大国库集中支付和非税收入收缴范围。

（九）加强“两型社会”建设，创造生态文明新优势。加快资源节约型、环境友好型社会建设，让玉林的天更蓝、地更绿、水更清、空气更清新，全力创建生态文明示范市。

加大节能减排工作力度。严格水资源管理，推进节水型社会建设，今年万元工业增加值用水量下降6%。促进土地、矿山资源的节约集约利用。抓好重点领域、重点行业节能减排项目建设，加强能源消费监管。加快淘汰立窑水泥、小造纸等落后产能，实现规模以上万元工业增加值能耗下降5.2%。

大力发展循环经济。进一步提高固体废弃物综合利用率。大力推进四家自治区综合利用企业、两家自治区循环经济示范企业建设，在建材、林化、制糖、冶金等行业构建循环经济产业链。加快龙潭再生资源加工利用园区建设，打造循环经济示范园区。

全面加强城乡生态建设。加强饮用水源、地表水或江河湖泊水源、大气污染、环境噪声、固体废物、危险废物、医疗废物、危险化学品、有机毒物、电磁辐射、机动车尾气和重金属污染综合整治，确保城乡环境安全。开展农村面源污染综合治理和农村环境连片整治，完善农业废弃物集中处理和农村生活垃圾、污水处理设施，加强农村畜禽养殖污染防治。

全力创建国家森林城市。深入实施“绿满八桂”造林绿化工程，大力开展全民植树造林活动，积极推进城镇绿化、通道绿化、村屯绿化和山上绿化，力争完成植树造林20万亩。积极做好申报国家森林城市工作。

（十）大力发展先进文化，促进文化软实力有新提升。传承好、发展好底蕴深厚的玉林文化，全面推动文化大发展大繁荣，全力创建岭南特色文化示范市。

抓好精神文明体系建设。加强社会公德、家庭美德、职业道德和未成年人思想道德建设。大力推进廉政文化建设。市财政安排400万元作为原始资金，设立好人好报基金，建立好人有好报的长效帮扶机制，使好人有好报的社会正气和价值导向成为统筹城乡发展、推进富民强市、建设幸福和谐玉林的强大精神力量。

全面发展公益性文化事业。加快市文化艺术中心、市博物馆建设，推进市图书馆新馆、群众艺术馆新馆、市工人文化宫的前期工作；抓好县级文化馆、图书馆改扩建工程，推进116个村级公共服务中心和299个“农家书屋”、355个“职工书屋”建设，继续实施广播电视“村村通”工程，加快构建覆盖城乡的公共文化服务体系。深入挖掘玉林特色的岭南文化内涵，推进文艺精品创作工程，设立文艺创作政府奖，实施签约文艺家制度。

加快发展文化产业。加快文化体制改革创新，筹建玉林文化产业集团，推进文化产业园和文化产业基地规划建设，培育发展文化产业集群。加快文化产业结构调整，壮大包装装潢、出版发行、印刷广告、演艺娱乐、影视制作、体育健身等传统文化产业，发展文化创意等新兴文化产业，培育文化休闲娱乐产业。

（十一）全面提升公共服务，确保群众生活水平有新提高。坚持民生优先，积极发展各项社会事业，不断提升公共服务水平，使广大人民群众生活更加幸福安康。

千方百计扩大就业。实施就业优先战略，实现经济增长与扩大就业良性互动。重点抓好高校毕业生、进城务工和就业困难人员等重点群体的就业工作。加快职业培训体系建设。建立城乡一体的就业创业服务体系和人力资源市场。

完善社会保障体系。扩大社会保险覆盖面。做好养老保险关系转移接续工作。加快社会保险信息化建设，推进五项社会保险“一卡通”。加强社会保险费征缴和社保基金监管。提高企业退休人员基本养老金水平。完善社会救助和保障标准与物价上涨挂钩联动机制。加快以居家养老为主、机构养老为补充的养老服务体系建设。

优先发展教育事业。实施教育布局结构调整规划。推进学前教育三年行动计划。深入开展城区中小学校建设大会战,玉林城区新建2所中小学校，县（市、区）新建5所以上中小学校。推进189个中小学校舍安全工程项目，全面完成第一轮建设任务。加强薄弱学校改造。推进特殊学校建设。

加快科技创新步伐。全面实施科技“十二五”规划和新一轮科技创新计划，启动知识产权战略推进工程。围绕千百亿元产业发展和地方重大科技需求，布局和建设一批重大科技创新平台和基地。加快推进清华科技园玉林分园规划建设。

加强人才队伍建设。深化“人才强市”战略，进一步完善人才培养、引进、选用机制和激励机制，深入实施高端人才引领工程、人才小高地建设提升等人才工程，完善玉林学者、特聘专家等人才制度，加强人才服务，统筹抓好各类人才队伍建设。

发展公共卫生事业。健全基层医疗卫生机构补偿和绩效考核机制。推进市骨科医院和5个乡镇卫生院、2个精神病医院、1个全科医生培养基地、3个县级急救中心、5个卫生监督所建设，加快完善基层医疗卫生服务体系。

抓好人口与计生工作。全面推进诚信计生。继续稳定低生育水平。加大综合治理人口性别比偏高工作力度。深入推进婚育新风进万家活动、关爱女孩行动和新农村新家庭计划。

加大扶贫开发力度。实施贫困村整村推进扶贫开发，优先在贫困地区安排资源开发和深加工项目，培育壮大贫困地区特色优势产业。实施职业培训工程和贫困地区“雨露计划”扶贫培训，提高贫困地区劳动力就业率。做好机关及企事业单位定点扶贫和对口支援工作。

做好物价调控工作。加强生产供给，增加大宗商品储备，健全调控和应急保供预案。抓好市场建设，增加农产品直销点，降低流通成本。完善价格调节基金制度，加强市场价格监督检查。

办实办好十项惠民工程。实施财政向民生倾斜政策，筹措资金51.24亿元，推进十项惠民工程，让发展成果惠及全市人民。

一是实施社保惠民工程。筹措资金7.7亿元，实现城乡居民基本养老保险、基本医疗保险制度全覆盖，其中2012年1月1日起实现新农保制度全覆盖；为5万城镇低保对象、45万农村低保对象发放最低生活保障补助；新建130个五保村。

二是实施卫生惠民工程。筹集资金15.87亿元，新农合筹资标准提高到每人290元，财政对新农合补助提高到每人240元，农民参合率巩固在90%以上；深入实施母婴健康“一免二补”幸福工程和地中海贫血防治计划。

三是实施教育惠民工程。筹措资金2.5亿元，用于扩大农村学前教育资源、农村义务教育阶段家庭困难寄宿生生活费补助、农村义务教育阶段学校维修改造、普通高中国家助学金等项目，确保每一个孩子不因家庭经济困难而失学。

四是实施强基惠民工程。筹措资金5.58亿元，实现新增70个行政村通硬化路，修建里程262公里；修建屯级道路824条516公里，其中贫困村屯级道路700条366公里；加大对2个小型农田水利重点县建设投入，治理河道5公里，完成29座小型病险水库除险加固，解决36.6万农村居民饮水安全问题。

五是实施安居惠民工程。筹措资金15亿元，建设保障性住房9800套。以解决农村中小学教师住房难为重点，加快公共租赁房、廉租房、经济适用房、限价商品房建设。改造农村危房8000户。

六是实施土地整治惠民工程。筹措资金1.86亿元,实施土地整理项目7个,对35个行政村的7.4万亩土地内的田、水、路、林、村进行综合整理。继续推进农村土地综合整治试点工作。

七是实施农补惠民工程。筹措资金1.23亿元，用于粮食作物良种补贴、农资综合补贴、农机具购置补贴等项目。

八是实施生态惠民工程。筹措资金9722万元，实施“绿满八桂”工程、森林生态效益补偿项目等，建设1.6万座沼气池；推进农村环境连片综合整治项目建设。

九是实施文化惠民工程。筹措资金3790万元，建设116个村级公共服务中心、299个“农家书屋”，实施农村电影公益性放映、20户以下自然村通广播电视工程等项目。

十是实施计生惠民工程。筹措资金1500万元，加大诚信计生奖扶力度，建设市计划生育服务中心，推进县、乡计划生育服务站所规范化建设和村级人口计生健康服务室、新家庭文化屋建设。

（十二）加强和创新社会管理，开创社会和谐稳定新局面。统筹第一要务和第一责任，加快提高社会管理科学化水平，深化和谐建设，全力创建社会管理创新示范市。

创新社会管理。提高全民法制意识。完善城乡社区自治和服务组织建设，健全基层管理服务体系。推进基层民主和基层政权建设，完善政务、村务、厂务公开制度。

强化安全生产责任。完善安全生产监管机制，抓好安全生产专项整治，切实减少安全生产事故。完善食品药品安全综合协调机制，落实食品药品安全责任制，提高食品药品安全保障水平。

切实维护社会稳定。完善市、县、镇、村四级综治信访维稳网络。加强社会稳定风险评估机制建设。加大人民调解力度，推进行业性纠纷调解中心建设。深入开展“大排查、大接访、大调处、大防控”活动。大力推进平安玉林建设，加强政法队伍建设，完善社会治安防控体系，抓好社会治安综合治理，加大对黑恶势力、“两抢一盗”、危害食品药品安全等违法犯罪活动的打击力度，切实增强人民群众的安全感。

做好支持部队和国防建设工作。大力推进双拥共建活动，巩固和发展军政军民团结。
